# Hebephrenic schizophrenia as a variant of frontotemporal dementia – the true dementia praecox?

**DOI:** 10.1192/j.eurpsy.2021.440

**Published:** 2021-08-13

**Authors:** A.B. Medeiros, N. Descalço, C. Fernandes Santos, R. Gomes, M. Veiga Pereira

**Affiliations:** Psychiatry And Mental Health Department, Hospital Garcia de Orta, E.P.E., Almada, Portugal, Portugal

**Keywords:** frontotemporal dementia, schizophrénia, Dementia praecox, hebephrenia

## Abstract

**Introduction:**

Frontotemporal Demential (FTD) is a neurodegenerative disorder evolving the frontal or temporal brain lobes. They have been described six variants. Behaviour variant (BvFTD) is the most common, and is characterized by changes in social behaviour and conduct, with loss of social awareness and poor impulse control. Hebephrenic schizophrenia (HSz), or disorganized schizophrenia, was recognized as a schizophrenia subtype, characterized by desorganized behaviour and a cognitive deteriorization. Subtypes of schizophrenia are no longer recognized as separate conditions neither in the Diagnostic and Statistical Manual of Mental Disorders, nor in the new International Statistical Classification of Diseases.

**Objectives:**

To review the literature about the concepts of hebephrenic schizophrenia and their similarities with the concept of frontotemporal dementia

**Methods:**

Narrative review of the literature on PubMed/MEDLINE, using the keywords “hebephrenic szchizophrenia” AND “frontotemporal dementia”. Only articles in English were included.

**Results:**

Some authors described dificulty in establish a diferential diagnosis between HSz and BvFTD. HSz has an earlier onset. However, BvFTD is an early age dementia. The fenomenology of both diseases is similar, and schizophrenia was historical conceptualized as praecox dementia. Frontotemporal abnormalities are common neuroimagiological findings in schizophrenia. Clinically, FTD shows a profound alteration in personality and social conduct, emotional blunting and loss of insight. Memory, intellectual functions, executive and attentional abilities may be disturbed in both.

**Conclusions:**

A diferential diagnosis between HSz and BvFTD is dificult to establish (clinically and imagiologically). The response to treatment is weak in both. It should be investigated the possibility they could be the same syndrome, onseting in diferent ages.

**Disclosure:**

No significant relationships.

